# Transcription Levels of nicotinamide nucleotide transhydrogenase and Its Antisense in Breast Cancer Samples 

**DOI:** 10.22074/cellj.2019.6238

**Published:** 2019-06-15

**Authors:** Soraya Saleh Gargari, Mohammad Taheri, Vahid Kholghi Oskooei, Mir Davood Omrani, Soudeh Ghafouri-Fard

**Affiliations:** 1 Feto-Maternal Unit, Shohadaye Tajrish Hospital, Shahid Beheshti University of Medical Sciences, Tehran, Iran; 2 Urogenital Stem Cell Research Center, Shahid Beheshti University of Medical Sciences, Tehran, Iran; 3 Department of Medical Genetics, Shahid Beheshti University of Medical Sciences, Tehran, Iran

**Keywords:** Breast Cancer, Long Non-Coding RNA, NNT

## Abstract

**Objective:**

To evaluate association of patients’ clinicopathological data with expression of nicotinamide nucleotide transhydrogenase (NNT) and naturally occurring antisense RNA of the same gene locus (NNT-AS1) in breast cancer samples.

**Materials and Methods:**

In the current case-control study, mean expressions of NNT and NNT-AS1 were assessed in 108 breast tissue samples including 54 invasive ductal carcinoma samples and 54 adjacent non-cancerous tissues (ANCTs) by quantitative reverse transcription-polymerase chain reaction (qRT-PCR).

**Results:**

NNT expression was not significantly different between tumor tissues and ANCTs. However, NNT-AS1 expression was significantly down-regulated in tumor tissues compared to ANCTs (expression ratio=0.51, P=0.01). NNT-AS1 expression was significantly higher in estrogen receptor (ER) negative samples, in comparison with ER positives (P=0.01). No considerable difference was found in the gene expressions between other subcategories of patients. Considerable correlations were detected between expression levels of these two genetic loci in both tumor tissues and ANCTs.

**Conclusion:**

In the current study, for the first time we simultaneously assessed expression of NNT and NNT-AS1 in breast cancer tissues. This study highlights association of ER status with dysregulation of NNT-AS1 in breast cancer tissues. Future researches are necessary to explore the function of this long non-coding RNA (lncRNA) in the pathogenesis of breast cancer.

## Introduction

Breast cancer, as the most frequent type of female cancer, has prompted several investigators to find diagnostic or prognostic biomarkers among long noncoding RNAs (lncRNAs) (1). This significant fraction of human transcriptome contributes to several aspects of cell physiology; so that dysregulation of them leads to development of cancer (2). An important subgroup of lncRNAs includes natural antisense transcripts (NATs) which are transcribed from the opposite DNA strand in relation to the sense transcripts, so their nucleotide sequences are complementary to the protein coding mRNA and the latter molecule is regulated by them. NATs can either inhibit or activate expression of the sense transcript (3).

Recent studies have highlighted contribution of *Nicotinamide nucleotide transhydrogenase (NNT)* and the related NAT in the pathogenesis of some human cancer types. NNT has an indispensible role in the homeostasis of NADH and NADPH (4). The significant contribution of NNT in mitochondrial antioxidant pathways shields cells from oxidative stress (5). *NNT* silencing decreases ability of cancer cells to preserve NAD+ and NADPH levels and suppresses their proliferation and aggressive behavior possibly via alteration of HIF-1α and HDAC1-dependent pathways (4). The related antisense transcript (*NNT-AS1*) participates in proliferation, migration, invasion and metastasis of colorectal cancer (CRC) cells (6). In hepatocellular carcinoma (HCC), *NNT-AS1* expression has enhanced cell proliferation and inhibited cycle arrest as well as apoptosis through modulation of *miR- 363*/CDK6 axis (7).

NNT loss has led to accumulation of acetylated catabolic substances of polyamines and a subsequent diminution of spermine and spermidine. Polyamine catabolism would mutually be elicited by oxidative stress and over-produce hydrogen peroxide, resulting in a malicious cycle that accelerates reactive oxygen species (ROS) production (8, 9). In spite of the appreciated role of these processes in pathogenesis of breast cancer (8), no study has yet assessed the simultaneous significance of NNT and NNT-AS1 dysregulation in breast cancer. Consequently, we conducted current study to assess expression of NNT and *NNT-AS1* in breast cancer tissues compared to ANCTs in association with patients’ clinicopathological data, to find whether their transcript levels are altered in breast cancer in parallel, or they can be used as biomarkers of breast cancer.

## Materials and Methods

### Patients

For the current case-control study, a total of 108 breast tissue samples -including 54 invasive ductal carcinoma samples and 54 ANCTs- were excised during surgery from patients hospitalized in Farmanieh and Sina Hospitals (Tehran, Iran) during January 2017-January 2018. Patients with definite diagnosis of invasive ductal carcinoma were included in the study. Those with other types of breast cancer and those received chemo-/radio-therapy before surgery were excluded from the study. All patients signed the written informed consent forms. The study protocol was permitted by the Ethical Committee of Shahid Beheshti University of Medical Sciences (IR.REC. SBMU.1397.764), Iran. Clinical and demographical data of patients were collected through evaluation of medical reports and interviews with patients.

### Expression analysis

Total RNA was extracted from tumor tissues and ANCTs using the TRIzol™ reagent (Invitrogen, USA) based on the company guidelines. In brief, we homogenized 75 mg of tissues in 1 ml TRIzol™ reagent, followed by RNA precipitation using isopropanol and washing it in 75% ethanol (10). After assessment of quality and quantity of the isolated total RNAs, a proportion of each RNA sample was converted to cDNA, using the RevertAid First Strand cDNA Synthesis Kit (TaKaRa, Japan). Transcript levels of NNT and NNT-AS1 genes were compared between tumor tissues and ANCTs using rotor gene 6000 Real-Time PCR System (Corbett Research, Australia). TaqMan Fast Universal PCR Master Mix (Applied Biosystems, USA) was used for the expression study. The Hypoxanthineguanine phosphoribosyl transferase (HPRT) gene was used for normalization of the gene and lncRNA expressions. The nucleotide sequences of primers are as follow:

HPRT1- F: 5ˊ-AGCCTAAGATGAGAGTTC-3ˊ,
  R: 5ˊ-CACAGAACTAGAACATTGATA-3ˊ,
  FAM-CATCTGGAGTCCTATTGACATCGC-TAMRA;
NNT1- F: 5ˊ-AGCCACCTTCTGTGTTACTTGC-3ˊ,
  R: 5ˊ-TAGCCCAGAGCTGCCATGAC-3ˊ,
  FAM-TCAACCGTCAGGCTGCCACTGCTG-TAMRA
NNT-AS1- F: 5ˊ-CTTCCACTCTCGGGGACAGG-3ˊ,
  R: 5ˊ-GCACCAGGTTTGATTGACAAGG-3ˊ,
  FAM-TTGTCTCTGCCTCGGCCTGCGG-TAMRA.

PCR efficiency and threshold cycle (Ct) values were obtained to quantify relative expression of each genetic locus in tumor tissues and ANCTs.

### Statistical analysis

SPSS software version 18.0 (SPSS Inc., USA) was used for statistical analysis. Ct values obtained from qRT-PCR experiments were adjusted based on the PCR efficiency values. Association of patients’ data with relative expression of the gene or lncRNA (down-/ up-regulation in the tumor samples vs. ANCTs) was evaluated using Chi-square test. Relative expression of each genetic loci in each tumor sample was calculated using EfficiencyCt reference/ EfficiencyCt target formula. Data is presented as mean ± SD. The difference in these values between individual groups of patients was evaluated using Tukey’s honest significance test. The pairwise correlation between relative transcription levels of the genetic loci in each set of samples (tumor tissues and ANCTs) was calculated using the regression model. For all statistical analyses, P<0.05 was regarded as significant. The receiver operating characteristic (ROC) curve was plotted to assess the power of genetic loci expression levels for diagnosis of disease status in breast samples. The Youden index (j) was applied to get the highest difference between sensitivity (true-positive rate) and 1-specificity (falsepositive rate).

## Results

### Overall demographic and clinical information of patients

Demographic and clinical information of the study participants are reported in Table 1.

### Transcript levels of NNT and NNT-AS1 in tumor tissues and ANCTs

**Fig.1:**
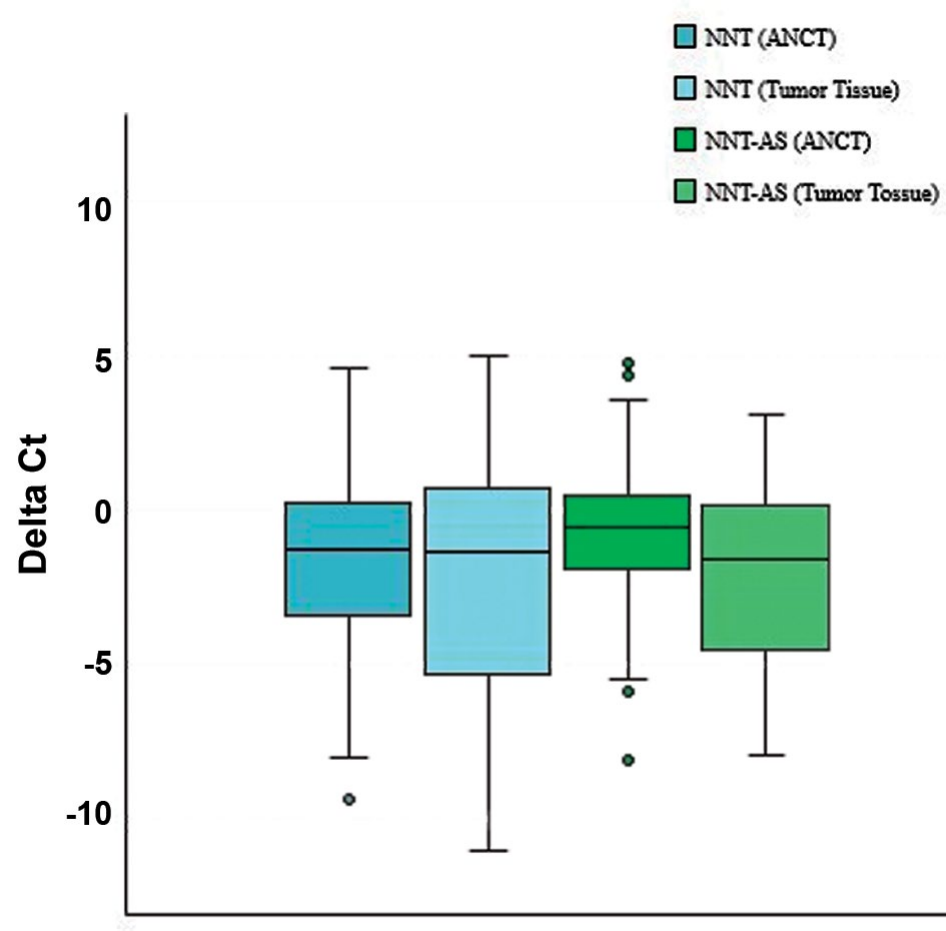
Relative transcription of *NNT* and *NNT-AS1* in tumor tissues (n=54) and ANCTs (n=54). *NNT*; Nicotinamide nucleotide transhydrogenase, *NNT-AS1; Nicotinamide nucleotide transhydrogenaseantisense 1*, ANCTs; Adjacent non-cancerous tissues, and ct; Threshold cycle.

*NNT* expression level was not significantly different between tumor tissues and ANCTs. However, *NNT-AS1* expression was significantly down-regulated in tumor tissues compared to ANCTs (expression ratio=0.51, P=0.01). Figure 1 shows relative expression of *NNT* and *NNT-AS1* in each set of samples, as described by -ΔCt values (Ct _reference_-Ct _target_).

**Table 1:** General demographic and clinical data of patients


Variables		Values

Age (Y)		51.79 ± 13.54 (29-81)
Menarche age (Y)		13 ± 1.65 (10-18)
Menopause age (Y)		44.91 ± 14.91 (38-60)
First pregnancy age (Y)		18.04 ± 8.36 (14-32)
Breast feeding duration (months)		41.62 ± 34.1 (3-120)
Positive family history for other cancers		17
Cancer stage		
	I	30.8
	II	28.8
	III	30.8
	IV	9.6
Overall grade		
	I	17
	II	49
	III	34
Mitotic rate		
	I	45.2
	II	42.9
	III	11.9
Tumor size		
	<2 cm	32
	≥2 cm, <5cm	66
	≥5 cm	2
Estrogen receptor		
	Positive	87.8
	Negative	12.2
Progesterone receptor		
	Positive	77.1
	Negative	22.9
HER2/Neu expression		
	Positive	25
	Negative	75


Data are presented as mean ± SD (range) or %.

### Association between relative expression of genetic loci and patients’ clinicopathological information

Based on the transcriptions in each tumor sample compared to related ANCT (<1 or >1), we categorized patients to up-/down-regulated groups. Then, we evaluated associations between such values and patients’ clinicopathological data. No association was found between relative expression of genetic loci in tumor tissue vs. ANCT and any of tumor characteristics (Table 2).

We also calculated relative expression of each genetic loci in tumor samples using Efficiency^Ct reference^/ Efficiency^Ct target^ formula and compared these values between tumor subgroups. We detected significantly higher expression of *NNT-AS1* in ER negative samples compared to ER positive cases (P=0.01). No remarkable difference was found between transcription levels of the other patient subcategories (Table 3).

### Correlations between transcript levels of *NNT* and *NNT-AS1* in tumor tissues and ANCTs

**Fig.2:**
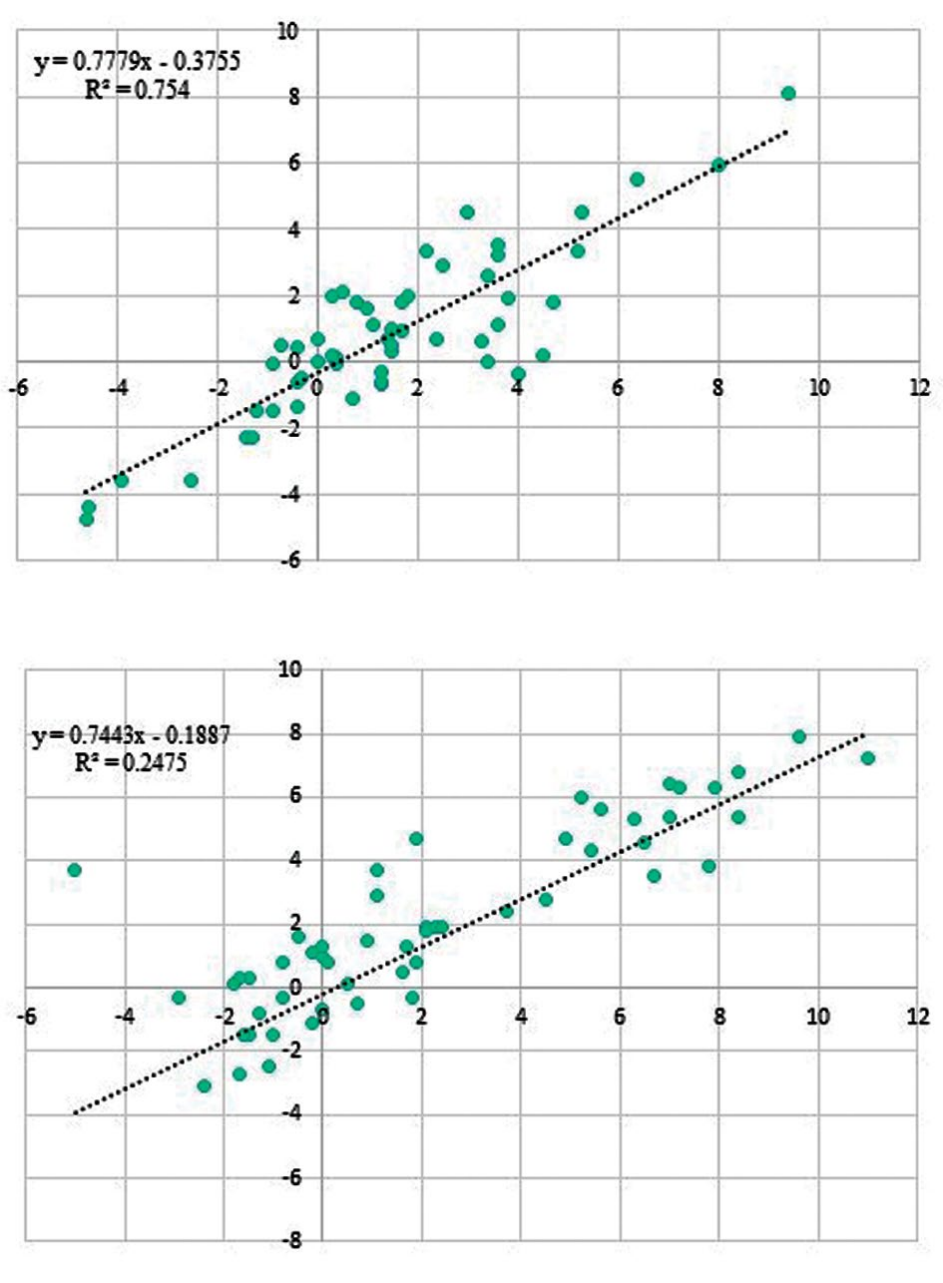
Correlation of *NNT* and *NNT-AS1* transcription levels. **A.** Shows the correlation in ANCTs and **B.** Shows the correlations in tumor tissues. *NNT;* Nicotinamide nucleotide transhydrogenase, *NNT-AS1;* Nicotinamide nucleotide transhydrogenase- antisense 1, and ANCTs; Adjacent noncancerous tissues.

Correlations between transcript level of NNT and its naturally occurring antisense was assessed in both tumor and ANCT samples. Transcription levels of *NNT* were correlated with the expression of NNTAS1 in both ANCT and tumor samples (Fig .2A, B, respectively). Considering R2 values, the correlation was stronger in ANCTs compared to tumor tissues.

**Table 2:** Association of relative transcriptions in tumor tissues compared to ANCTs, with patients’ clinicopathological data


Parameters		NNTUp-regulation	NNTDown-regulation	P value	NNT-AS1Up-regulation	NNT-AS1Down-regulation	P value

Age				0.4			0.79
	<55 Y	15 (45.5)	18 (54.5)		12 (35.3)	22 (64.7)	
	≥55 Y	6 (33.3)	12 (66.7)		7 (38.9)	11 (61.1)	
Stage				0.9			0.76
	I	6 (37.5)	10 (64.5)		5 (33.3)	10 (66.7)	
	II	5 (35.7)	9 (64.3)		7 (46.7)	8 (53.3)	
	III	7 (46.7)	8 (53.3)		5 (56.3)	11 (43.7)	
	IV	2 (50)	2 (50)		1 (25)	3 (75)	
Histological grade				0.38			0.97
	I	5 (62.5)	3 (37.5)		3 (37.5)	5 (62.5)	
	II	8 (34.8)	15 (65.2)		8 (36.4)	14 (63.6)	
	III	5 (37.5)	8 (62.5)		5 (33.3)	10 (66.7)	
Mitotic rate				0.57			0.26
	I	8 (42.1)	11 (57.9)		9 (50)	9 (50)	
	II	5 (33.3)	10 (66.7)		4 (23.5)	13 (76.5)	
	III	3 (60)	2 (40)		2 (40)	3 (60)	
Tumor size				0.67			0.31
	<2	7 (43.8)	9 (56.2)		7 (43.8)	9 (56.2)	
	2-5	12 (38.7)	19 (61.3)		10 (32.3)	21 (67.7)	
	>5	0 (0)	1 (100)		1 (100)	0 (0)	
ER status				0.84			0.33
	Positive	17 (40.5)	21 (58.3)		15 (36.6)	26 (63.4)	
	Negative	2 (40)	3 (60)		1 (16.7)	5 (83.3)	
PR status				0.92			0.54
	Positive	15 (41.7)	21 (58.3)		13 (37.1)	22 (62.9)	
	Negative	4 (40)	6 (60)		3 (27.3)	8 (72.7)	
HER2 status				0.51			0.54
	Positive	4 (33.3)	8 (66.7)		3 (27.3)	8 (72.7)	
	Negative	15 (44.1)	19 (54.3)		13 (37.1)	22 (62.9)	


*NNT; Nicotinamide nucleotide transhydrogenase, NNT-AS1;Nicotinamide nucleotide transhydrogenase-antisense 1,* ANCTs; Adjacent noncancerous tissues, ER; Estrogen receptor, PR; Progesterone receptor, and HER2; Human epidermal growth factor receptor 2.

**Table 3:** Association of transcription levels in tumor tissues with tumors characteristics (mean ± SD values of EfficiencyCt reference- EfficiencyCt target arepresented)


Parameters		NNT	P value	NNT-AS1	P value

Age					
	<55 vs. ≥55 Y	71.97 (221.19) vs. 2.6 (4.63)	0.18	33.96 (127.22) vs. 2.79 (4.94)	0.29
ER status					
	ER(+) vs. ER(-)	54.76 (198.15) vs. 20.84 (45.58)	0.68	10.58 (39.1) vs. 119.63 (289.12)	0.01
PR status					
	PR(+) vs. PR(-)	63.53 (212.69) vs. 11.72 (33.89)	0.42	12.23 (42) vs. 57.81 (213.71)	0.15
HER2 status					
	HER2 (+) vs. HER2 (-)	1.17 (2.78) vs. 68.49 (215.27)	0.28	1.53 (4.68) vs. 32.06 (123.7)	0.4
Tumor grade					
	Grade I vs. II	84.71 (222.77) vs. 28.58 (131.89)	0.76	5.61 (7.02) vs. 13.89 (52.76)	0.98
	Grade I vs. III	84.71 (222.77) vs. 75.51 (253.41)	0.99	5.61 (7.02) vs. 53.38 (182.01)	0.58
	Grade II vs. III	28.58 (131.89) vs. 75.51 (253.41)	0.74	13.89 (52.76) vs. 53.38 (182.01)	0.53


*NNT; Nicotinamide nucleotide transhydrogenase, NNT-AS1; Nicotinamide nucleotide transhydrogenase-antisense 1,* ct; Threshold cycle, ER; Estrogen receptor, PR; Progesterone receptor, and HER2; Human epidermal growth factor receptor 2. Data are presented as mean (SD) values.

### Receiver operating characteristic curve analysis

**Fig.3:**
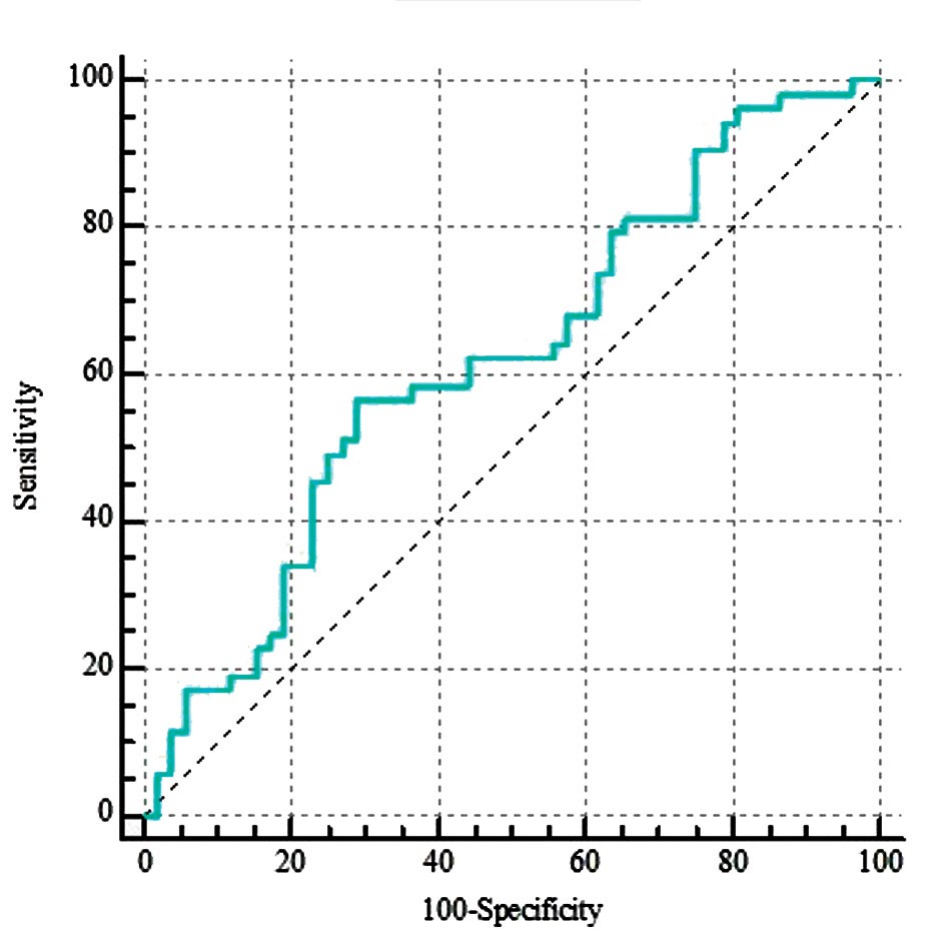
Results of receiver operating characteristic (ROC) curve assessment analysis for NNT-AS1 performance in breast cancer diagnosis. The values were analysed based on Efficiency^Ct reference^ - Efficiency^Ct target^ .

The power of *NNT-AS1* expression in prediction of disease status in breast samples was evaluated using ROC curve (Fig .3). Assessment of this lncRNA transcription level shows 71.2% specificity and 56.6% sensitivity for breast cancer diagnosis.

## Discussion

In the current study, for the first time we simultaneously assessed expression of *NNT* and *NNT-AS1* in breast cancer tissues in comparison with ANCTs and found downregulation of *NNT-AS1* in tumor tissues, in spite of detecting similar level of *NNT* expression in tumor tissues and ANCTs. *NNT-AS1* has previously been shown to exert oncogenic effects in CRC, HCC, osteosarcoma and cervical cancer (6, 7, 11, 12). However, expression of this lncRNA was significantly decreased in patients with ovarian cancer as well as the human ovarian cancer cell lines. Moreover, in vitro studies has shown that *NNT-AS1* silencing enhances cell migration and invasion, while it suppresses apoptosis (13). So, the observed down-regulation of *NNT-AS1* in the current study is consistent with the previously reported dysregulation of this molecule in ovarian cancer. This lncRNA is transcribed in the opposite direction of *NNT* gene and it has no intersection with latter gene nucleotide sequence (14). Most recently, Li et al. (14) demonstrated overexpression of *NNT-AS1* in breast tumor tissues compared to ANCTs in correlation with patients’ survival and HER2, but not ER, status. In vitro experiments showed that *NNT-AS1* contributes to breast cancer pathogenesis via altering miR-142-3p/ZEB1 axis. The inconsistency between our results and their results might be due to a possible difference in the mean age of the study participants or an ethnic-based modulator of transcription. They have shown that ZEB1 is positively regulated by *NNT-AS1*. In our previous study, on the same cohort of patients, we failed to demonstrate any significant change in ZEB1 expression between tumor tissues and ANCTs (15). So, we hypothesized that *NNT-AS1* might participate in the pathogenesis of breast cancer through other mechanisms, including regulation of *NNT* expression. Significant correlation between *NNT* and *NNT-AS1* expressions, especially in non-tumor tissues, implies the presence of a feed-forward loop between these two genetic loci which should be assessed in future studies.

In spite of totally down-regulation of *NNT-AS1* in tumor tissues compared to ANCTs, we demonstrated higher expression of it in ER negative tumor samples, compared to ER positive samples, likely suggesting the importance of this lncRNA in pathogenesis of ER negative breast cancers. Future studies are needed to evaluate expression of *NNT-AS1* in larger cohorts of patients with regards to hormone receptor status.

Finally, we evaluated the power of *NNT-AS1* expression in prediction of the disease status in breast samples. Although transcription level of this genetic locus is not individually a sensitive marker for prediction of breast cancer, it might increase specificity of other putative panels of gene expression.

## Conclusion

The current study shows down-regulation of *NNT-AS1* in breast cancer tissues compared to ANCTs in association with ER status. Future studies are necessary to explore function of this lncRNA in the pathogenesis of breast cancer
